# Supplementary material for: Blood Flow Restriction Training and Its Use in Rehabilitation After Anterior Cruciate Ligament Reconstruction: A Systematic Review and Meta-Analysis
Source: J Clin Med. 2024 Oct 20;13(20):6265. doi: 10.3390/jcm13206265 (PMC11508829; doi:10.3390/jcm13206265)
Supplement: Supplementary file 1 [file jcm-13-06265-s001.zip › jcm-3224127-supplementary.pdf]

Supplementary Materials

Blood Flow Restriction Training and its use in Rehabilitation after Anterior Cruciate Ligament Reconstruction: A Systematic Review and Meta-analysis

Jamaal Butt [1], and Zubair Ahmed [1, 2, *]

[1] Department of Inflammation and Ageing, School of Infection, Inflammation and Immunology, University of Birmingham, Edgbaston, Birmingham, B15 2TT, UK; jyb4@student.le.ac.uk

[2] University Hospitals Birmingham NHS Trust, Mindelsohn Way, Edgbaston, Birmingham, B15 2GW

[*] Correspondence: z.ahmed.1@bham.ac.uk

Table S1: Data collection table for study characteristics

To include:

- Study authors
- Year of publication
- Country of origin
- Type of occlusion used
- Study size (number of participants in both arms)
- Gender distribution across both arms
- Average age of participants
- Length of rehabilitation
- Length of follow up / dates at which participants had data recorded

Authors	Year Published	Country	Occlusion Type	N number	Gender	Participant Age/years	Length of rehabilitation/ weeks	Timing of data collection after initial evaluation/ weeks
Jack et al	2022	USA	Delfi Medical automated torniquet at 80% arterial limb occlusion pressure around proximal thigh	Control – 17<br>BFR - 15	Control – M7/F8<br>BFR – M12/F5	Control – 24.1 (+-7.2)<br>BFR – 28.1 (+-7.2)	12 weeks (biweekly sessions) (beginning within 7 days post op)	8- and 12-weeks from beginning training
Hughes et al	2019	UK	Delfi Medical automated torniquet at 80% arterial limb occlusion pressure around proximal thigh	Heavy Load Resistance Training – 14<br>BFR - 14	Heavy Load Resistance Training – M10/F2<br>BFR – M7/F5	Heavy Load Resistance Training – 29 (+-7)<br>BFR – 29(+-7)	8 weeks (biweekly sessions) (beginning at 2 weeks post op)	9 weeks from the beginning of training
De Melo et al	2022	Brazil	Cuff Scientific Leg® – WCS 80% arterial limb occlusion	Control – 12<br>BFR - 12	Control – M9/F3<br>BFR – M8/F4	Control – 39.6 (+-10.8)<br>BFR – 41.1 (+-9.8)	12 weeks (biweekly sessions)	4/8/12 weeks post op

			pressure around proximal thigh					
Li et al	2023	China	Air band Bluetooth pressurized device at 40% occlusion pressure and 80% occlusion pressure	Control – 6<br>40% BFR – 9<br>80% BFR - 8	Gender split across groups was not provided	18-40 years old but the specific split was not detailed	8 weeks (biweekly session) (beginning at least 8 weeks post op)	8 weeks from the beginning of training
Kacin et al	2021	Slovenia	BFR GROUP Double-chamber pneumatic cuff with asymmetric pressure Ischemic Trainer inflated to 150mmHg<br>SHAM BFR GROUP Double-chamber pneumatic cuff with asymmetric pressure Ischemic Trainer inflated to 20mmHg	Control – 6<br>20mmHg BFR – 6<br>150mmHg BFR - 6	Control – M3/F3<br>20mmHg BFR – M3/F3<br>150mmHg BFR – M3/F3	Control – 36(+-9)<br>20mmHg BFR – 38(+-8)<br>150mmHg BFR – 38(+-6)	3 weeks (3 sessions a week)	3 weeks from the beginning of training
Ohta et al	2003		Air torniquet at 180mmHg Worn around proximal thigh	Control – 22<br>BFR - 22	Control – M12/F20<br>BFR – M13/F9	Control – 30(+-9.7)<br>BFR – 28 (+-9.7)	16 weeks (6 times weekly with different exercises throughout the program)	16 weeks from surgery

Jung et al	2022	Korea	Not stated	Control – 12<br>BFR - 12	Control – M9/F3<br>BFR – M9/F3	Control – 27.83(+-8.43)<br>BFR – 30.83(+-7.59)	12 weeks beginning 3 days post op (3 times a week with different exercises throughout the program)	12 weeks and 2 days from the beginning of training
Iversen et al	2015	Norway	Delphi low pressure cuff with pressure starting at 130mmHg and rising to 180mmHg by 10th day of training	Control – 12<br>BFR - 12	Control – M7/F5<br>BFR – M7/F5	Control – 29.8(+-9.3)<br>BFR – 24.9(+-7.4)	14 days with occlusion if in intervention group + 2 days without stimulus Beginning at 2 days post-surgery<br>(2 times a day)	16 days post-surgery

Table S2: Data collection table for primary outcomes

To include:

- Author
- Number of patients in the control
- Number of patients in the intervention
- Primary outcome and how it was measured
- Primary outcome results for control group (mean and standard deviation)
- Primary outcome results for intervention group (mean and standard deviation)
- P value (actual p value if given)

Table S2.1 : Primary outcome of interest: Changes in femur/thigh muscle mass

Author	Control (N)	Intervention (R)	Way outcome was measured	Control Results	Intervention Results	P value
Jack et al (6 weeks)	17	15	DEXA scan of thigh, change in lean mass / kg	-0.27 (0.03)	-0.09 (0.03)	<0.01
(12 weeks)				-0.12 (0.05)	0.00 (0.03)	<0.01
Hughes et al	14	14	USS of vastus lateralis, change in muscle thickness/cm	1.94 (0.44)	1.91 (0.39)	0.85
De Melo et al	12	12	Not recorded	Not recorded	Not recorded	Not recorded
Li et al	6	40% BFR – 9<br>80% BFR - 8	Muscle thickness/cm	4.32 (0.7)	40% BFR – 5.23 (0.84)<br>80% BFR – 6.26 (0.64)	N vs 40% BFR <0.05 (0.017)<br>N vs 80% BFR <0.05 (0.000)<br>40% vs 80% <0.05 (0.004)
Kacin et al	6	20mmHg BFR – 6<br>150mmHg BFR - 6	Muscle size (cross sectional area of quadriceps)/mm2	Not reported	20mmHg BFR: -1.1(2.1%)<br>150mmHg BFR: 5.0 (3%)	20mmHg vs 150mmHg <0.01
Ohta et al	22	22				
Jung et al	12	12	Not recorded	Not recorded	Not recorded	Not recorded

Iversen et al	12	12	Cross sectional area of femur 40% from knee joint/cm2<br>Cross sectional area of femur 50% from knee joint/cm2	40%: - 9.2(0.8)<br>50%: - 11.5 (0.7)<br>Mean Change: - 13.1(1.0)	40%: -9.7(1.0)<br>50%: - 13.7(0.9)<br>Mean Change: - 13.8(1.1)	0.6265

Table S2.2 : Primary outcome of interest: Changes in Leg Strength (flexion and extension)

Author	Control (N)	Intervention (R)	Way outcome was measured	Control Results	Intervention Results	P value
Jack et al (8 weeks)	17	15	Single leg leg press/kg	58.6(8.0)	59.3(10.0)	Not reported as not significant
(12 weeks)				76.5(9.0)	72.7(8.7)	Not reported as not significant
Jack et al (8 weeks)			Single leg hamstring curl/kg	29.9(5.1)	28.2(4.2)	Not reported as not significant
(12 weeks)				38.5(4.5)	37.7(4.9)	Not reported as not significant
Hughes et al	14	14	Isokinetic strength 60°/s flexion	Unable to determine	Unable to determine	Unable to determine
			Isokinetic strength 60°/s extension	Unable to determine	Unable to determine	0.20
			Isokinetic strength 150°/s flexion	Unable to determine	Unable to determine	Raw values not provided
			Isokinetic strength 150°/s extension	Unable to determine	Unable to determine	<0.01
			Isokinetic strength 300°/s flexion	Unable to determine	Unable to determine	Unable to determine
			Isokinetic strength 300°/s extension	Unable to determine	Unable to determine	<0.01
De Melo et al (4 weeks	12	12	Knee extension/N	≈12	≈15	Not reported as not significant
			Knee flexion/N	≈7	≈8	Not reported as not significant

(8 weeks)			Knee extension/N	≈13	≈17	Not reported as not significant
			Knee flexion/N	≈8 (unable to determine s.d)	≈10 (unable to determine s.d)	<0.01
(12 weeks)			Knee extension/N	≈14 (unable to determine s.d)	≈20 (unable to determine s.d)	<0.01
			Knee flexion/N	≈9 (unable to determine s.d)	≈13 (unable to determine s.d)	<0.01
Li et al	6	40% BFR – 9<br>80% BFR - 8	Combined knee flexion and extension/N/Kg 60°/s	Before: 90.20 (21.53)<br>After: 94.05 (17.62)<br>Δ 3.58	40% BFR:<br>Before: 93.02 (46.61)<br>After: 123.94 (28.13)<br>Δ 30.92<br>80% BFR:<br>Before: 107.53 (46.75)<br>After: 152.04 (25.45)<br>Δ 44.51	N vs 40% BFR <0.05 (0.030)<br>N vs 80% BFR <0.05 (0.000)<br>40% vs 80% <0.05 (0.027)
			Combined knee flexion and extension/N/Kg 180°/s	Before: 70.98 (15.07)<br>After: 73.28 (15.73)<br>Δ 2.3	40% BFR:<br>Before: 74.72 (39.93)<br>After: 102.06 (17.34)<br>Δ 27.33<br>80% BFR:<br>Before: 78.69 (34.47)<br>After: 137.65 (25.54)<br>Δ 58.96	N vs 40% BFR <0.05 (0.013)<br>N vs 80% BFR <0.05 (0.000)<br>40% vs 80% <0.05 (0.001)
Kacin et al	6	20mmHg BFR – 6<br>150mmHg BFR - 6	Extensor muscles Peak torque 60 °/s (Nm)	Not reported	20mmHg BFR: 147 (52)<br>150mmHg BFR: 171 (64)	<0.05 (0.018)
			Flexor muscles Peak torque 60 °/s (Nm)	Not reported	20mmHg BFR: 97 (0.731)<br>150mmHg BFR: 117 (33)	0.731
			Extensor muscles Peak torque 120 °/s (Nm)	Not reported	20mmHg BFR: 121 (44)<br>150mmHg BFR: 139 (48)	<0.05 (0.027)

			Flexor muscles<br>Peak torque 120 °/s (Nm)	Not reported	20mmHg BFR: 84(20)<br>150mmHg BFR: 100 (24)	0.172
Ohta et al	22	22				
Jung et al	12	12	Extensor muscles<br>Peak torque 60 °/s (Nm)	Before: 121.70 (44.48)<br>After: 139.42 (48.06)<br>Δ 17.72	Before: 94.08 (35.89)<br>After: 131.08 (47.23)<br>Δ 37.00	0.279
			Flexor muscles<br>Peak torque 60 °/s (Nm)	Before :66.43 (22.90)<br>After: 72.10 (18.22)<br>Δ 5.67	Before: 53.17 (22.25)<br>After: 80.50 (23.73)<br>Δ 27.33	<0.05 (0.006)
Iversen et al	12	12	Not measured	Not measured	Not measured	Not measured

Table S3: Data collection table for secondary outcomes

To include:

- Author
- Number of patients in control
- Number of patients in intervention
- Secondary outcomes of the paper and how they were measured
- Secondary outcomes of control group (mean and standard deviation)
- Secondary outcome results of intervention group (mean and standard deviation)
- P value (actual P value if given)
- Using only Lysholm and IKDC as more specific to functioning and sports

Table S3.1 : Secondary outcome of interest: Lysholm Score

Author	Control (N)	Intervention (N)	Secondary outcomes	Control results	Intervention results	P value
Jack et al	17	15	Not Measured			
Hughes et al	14	14	Lysholm Score	Before: 111.83 (14.44)<br>After: 91.92 (14.42)	Before: 115.42 (16.58)<br>After: 93.93 (14.04)	Before: 0.58<br>After: 0.73
De Melo et al (4 weeks	12	12	Lysholm Score	81.75 (3.80)	92.00 (2.39)	
(8 weeks)				86.17 (1.95)	100.00 (0)	<0.05
(12 weeks)				90.58 (0,84)	100,00 (0)	<0.05
Li et al	6	40% BFR – 9<br>80% BFR - 8	Not measured			
Kacin et al	6	20mmHg BFR – 6<br>150mmHg BFR - 6	Not measured			
Ohta et al	22	22	Not measured			
Jung et al	12	12	Lysholm Score	Before: 69.17 (11.94)<br>After: 83.08 (6.32)<br>Δ 13.92	Before: 63.67 (21.82)<br>After: 86.50 (12.27)<br>Δ 22.83	0.158
Iversen et al	12	12	Not measured			

Table S3.2 : Secondary outcome of interest: IKDC score

Author	Control (N)	Intervention (N)	Secondary outcomes	Control results	Intervention results	P value
Jack et al	17	15	Not Measured			
Hughes et al	14	14	IKDC	Before: 48.33 (10.30)<br>After: 30.17 (9.31)	Before: 48.59 (16.71)<br>After: 32.03 (10.34)	Before: 0.96<br>After: 0.65
De Melo et al (4 weeks	12	12	IKDC	51.58 (2.02)	58.25 (1.50)	
(8 weeks)				55.00 (1.6)	65.08 (0.38)	<0.05
(12 weeks)				60.92 (1.59)	74.83 (0.44)	<0.05
Li et al	6	40% BFR – 9<br>80% BFR - 8	IKDC	Before: 58.50 (13.32)<br>After: 59.00 (10.79)<br>Δ 0.5	40% BFR:<br>Before: 57.44 (14.03)<br>After: 65.11 (10.65)<br>Δ 7.67<br>80% BFR:<br>Before: 55.75 (12.13)<br>After: 75.38 (10.45)<br>Δ 19.63	N vs 40% BFR 0.295<br>N vs 80% BFR <0.05 (0.000)<br>40% vs 80% <0.05 (0.001)
Kacin et al	6	20mmHg BFR – 6<br>150mmHg BFR - 6	Not measured			
Ohta et al	22	22	Not measured			
Jung et al	12	12	IKDC	Before: 62.36 (14.11)<br>After: 75.25 (9.94)<br>Δ 10.86	Before: 59.55 (12.04)<br>After: 76.42 (14.32)<br>Δ 16.87	0.290
Iversen et al	12	12	Not measured			
